# HER2-positive pure mucinous breast carcinoma: A case report and literature review

**DOI:** 10.1097/MD.0000000000020996

**Published:** 2020-08-14

**Authors:** Xingjuan Zhao, Xuan Yang, Runfang Gao, Liqin Zhai, Lizhu Yang, Keda Yu

**Affiliations:** a Department of Mammary Gland; b Department of Pathology, Shanxi People's Hospital, Taiyuan; c Department of Mammary Gland, Fudan University Cancer Hospital, Shanghai, China.

**Keywords:** estrogen receptor positive, HER2-positive, progesterone receptor negative, pure mucinous carcinoma

## Abstract

**Introduction:**

Pure mucinous carcinoma is a rare type of breast carcinoma, but it usually has a favorable prognosis. Tumors of pure mucinous carcinoma are typically positive for both estrogen receptor (ER) and progesterone receptor (PR), and they do not commonly overexpress human epidermal growth factor receptor 2 (HER2). However, when tumors have HER2 overexpression and are progesterone receptor negative, the prognosis is worse.

**Patient concerns:**

A 59-year-old female reported a slow growth mass of 3 years, which was radiologically diagnosed as fibroadenoma at another institution. The patient came to our institution for treatment and follow-up. She had no salient past history.

**Diagnosis:**

Excisional biopsy revealed a pure mucinous breast carcinoma that was ER (100%, moderate-strong intensity), PR(−), 5% Ki-67 (+), and HER2(3+) by immunohistochemistry. The *HER2* gene was found to be amplified by fluorescence in situ hybridization (FISH). The clinical staging was T2N0M0, with pathological grade I, subtype luminal B.

**Interventions:**

After a modified radical mastectomy, she received four 21-day cycles of intravenous docetaxel (75 mg/m^2^), intravenous cyclophosphamide (600 mg/m^2^), and intravenous trastuzumab (8 mg/kg) (loading dose) on day 1 followed by 6 mg/kg every 3 weeks to complete a full year of treatment. She then received 2.5 mg of letrozole daily for 5 years.

**Outcomes:**

After following up for 2 years, the patient's outcome was survival without recurrence. Cardiac ultrasounds were performed every 3 months and there was no change in the left ventricular ejection fraction (LEVF).

**Conclusion:**

It is essential to correctly diagnose the ER(+), PR(−) HER2(+) subtype in mucinous carcinoma. This type should be treated with chemotherapy and anti-HER2 therapy, as well as aromatase inhibitor endocrine therapy.

## 1 Introduction

Mucinous carcinoma is a rare histologic type of breast carcinoma that accounts for 1% to 6% of all breast cancers.^[1,2]^ According to the proportion of the mucinous component, mucinous breast cancer is divided into 2 types: pure and mixed. To be considered pure mucinous breast cancer, the mucinous component of the tumor must be at least 90%. Pure mucinous carcinoma of the breast accounts for only 0.8% of all breast cancers.^[3]^ Tumors most frequently occur in postmenopausal females from 31 to 88 years old (with an average age of 67 years).^[4]^ Pure mucinous carcinoma tumors are usually positive for estrogen and progesterone receptors, and they usually do not overexpress human epidermal growth factor receptor 2 (HER2).^[5]^ Compared to mixed mucinous breast cancer, it has a favorable prognosis.^[6]^ However, when HER2 is overexpressed, the prognosis is worse and trastuzumab treatment is typically indicated.^[7,8]^ Here, we report a positive HER2 patient with pure mucinous breast carcinoma. This case report has been described under the SCARE criteria.^[9]^

## 2 Case report

A 59-year-old Chinese female had a mass on her left breast for 3 years. The mass was radiologically diagnosed as fibroadenoma at another institution, and the patient came to our institution for treatment and follow-up. She had no salient past history.

The mass was 3×2 cm in size. Upon palpation, it was detected 1 cm above the nipple at the left breast, hard, and had a clear margin. An ultrasound was performed, showing a hypoechoic mass with circumscribed margins. Upon mammography, it appeared as a high density and round or oval mass with a well-defined margin. A core needle biopsy was performed, and the case presented positive for mucinous carcinoma. The clinical staging was T2N0M0. The patient selected modified radical mastectomy as treatment.

**Figure 1:**
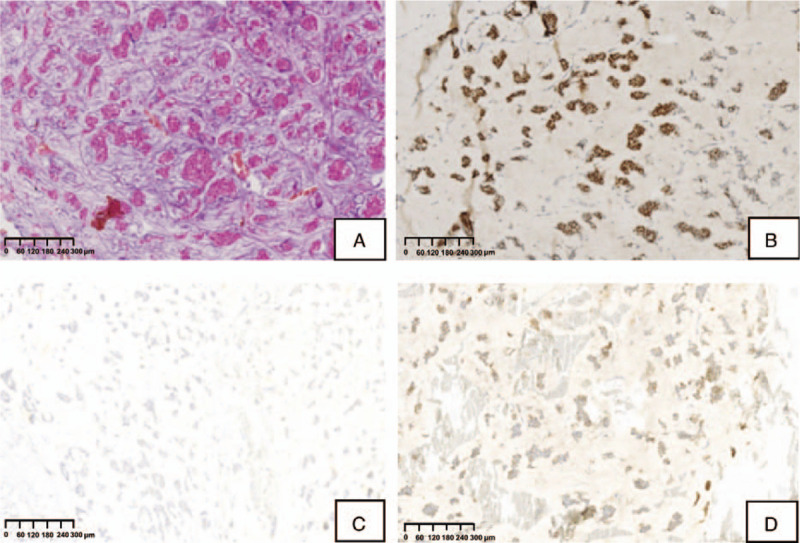
A, Carcinoma cells with a moderate amount of eosinophilic cytoplasm surrounded by lakes of extracellular matrix. B, ER was positive with 100% of carcinoma cells with moderate-strong intensity. C, PR was negative for carcinoma cells. D, Ki-67 was 5% of the carcinoma cells with proliferative activity. ER = estrogen receptor, PR = progesterone receptor.

**Figure 2:**
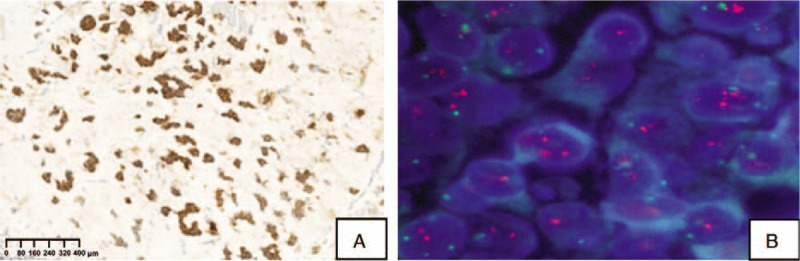
A, HER2 with 3+ of carcinoma cells. B, FISH: HER2 signals with clusters of positive. FISH = fluorescence in situ hybridization, HER2 = human epidermal growth factor receptor 2.

According to the postoperative pathology, we found that the tumor was composed of mucinous lakes with numerous tumor cells of medium-to-large size with a moderate amount of eosinophilic cytoplasm, confirming the presence of pure mucinous carcinoma (Fig. 1A). No perineural or vascular invasion was noted. Immunohistochemistry showed that tumor cells were 100% positive with moderate-strong intensity for estrogen receptor, negative for progesterone receptor, 5% Ki-67 positive (Fig. 1B–D) and had an HER2 score of 3+ (Fig. 2A). The *HER2* gene was amplified and detected by fluorescence in situ hybridization (FISH) (Fig. 2B). The pathological evaluation of the lymph nodes was negative for metastasis. The pathological size of the mass was 2×2 cm. The pathological grade was I, subtype luminal B.

Since the tumor was ER(+), PR(−), and HER2(+), the patient received four 21-day cycles of intravenous docetaxel (75 mg/m^2^), intravenous cyclophosphamide (600 mg/m^2^), and intravenous trastuzumab 8 mg/kg (loading dose) on day 1 followed by 6 mg/kg every 3 weeks to complete a full year of intravenous treatment. This was followed by 2.5 mg of letrozole per day for 5 years. After following up for 2 years, the patient's outcome was survival without recurrence. Adverse effect of grade 2 chemotherapy-induced neutropenia was observed. Cardiac ultrasounds were performed every 3 months and there was no change in the left ventricular ejection fraction (LEVF) noted.

This study was approved by the Ethics Committee of the Shanxi Provincial People's Hospital (No. [2019]27), and patient's consent was taken.

## 3 Discussion

Mucinous carcinomas represent 1% to 6% of invasive breast carcinomas. One typical pathological feature is the extracellular mucin within the tumoral cells. Mucinous carcinomas can be misdiagnosed as benign lesions because of their slow growth, clear boundary, and smooth border.^[3,10]^ Immunohistochemistry of these tumors shows that they are usually positive for estrogen receptor (94%) and progesterone receptor (80%), as well as negative for HER2 (93%).^[11–13]^ This indicates a favorable prognosis for the disease.^[13,14]^

We present a case of a pure mucinous carcinoma in a 59-year-old Chinese female. This case is unusual in being a low-frequency malignancy that is HER2-positive, ER-positive, and PR-negative, which has rarely been reported in the literature. Due to the low HER2-positive rate of mucinous carcinoma, NCCN guidelines^[15]^ showed endocrine therapy for axillary lymph node negative, ER-positive, and/or PR-positive mucinous carcinoma. In fact, endocrine therapy is only recommended for HER2-negative mucinous carcinoma. For HER2-positive mucinous carcinoma, the guidelines do not make a clear recommendation.

There are several issues to consider when developing an appropriate treatment regimen for a case such as this one. First, ER(+), PR(−) breast cancer is a highly invasive type. The lack of PR is correlated with low concentrations of serum estrogen, low levels of nuclear ER, and crosstalk between HER2 and membranous ER. ER(+), PR(−) breast cancers usually have higher levels of HER2 than ER(+), PR(+) tumors.^[16]^ ER(+), PR(−) tumors overexpress HER2 in 21% of cases compared to 14% of ER(+), PR(+) tumors.^[17]^

Second, HER2 positivity is an independent predictor of poor prognosis for breast cancer. HER2-positive breast cancer often requires anti-HER2 therapy. A number of clinical trials have confirmed the effectiveness of anti-HER2 therapy, such as the HERA, NCCTG N9831, NSABP B-31, BCIRG 006, and TC4H trials.^[18]^ The Neo Sphere trial demonstrated that anti-HER2 therapy should be administered in conjunction with chemotherapy for the best treatment effect.^[19]^ Thus, for HER2-positive breast cancer, chemotherapy is as indispensable as anti-HER2 therapy.

Third, the complex crosstalk between HER2 and membranous ER may cause tamoxifen resistance.^[16,20]^ Therefore, aromatase inhibitors, fulvestrant^[20]^ and chemotherapy may be the most appropriate treatments for HER2-positive breast cancer. A meta-analysis^[21]^ showed an association between *HER2* gene amplification and the failure of endocrine therapy for metastatic breast cancer. Endocrine therapy alone should not be considered a first choice for patients with ER(+), HER2-positive metastatic breast tumors. Instead, for this type of patient, the treatment should involve chemotherapy alone or in combination with trastuzumab.

This patient has a low Ki-67. A lower Ki-67 index has been correlated with good prognosis and late recurrence. Thus is important to take Ki-67 into account in breast cancer treatment and follow-up.^[22,23]^ It may serve as an additional predictor of survival in luminal B node negative breast cancer.^[23]^

Although there are some reports^[24,25]^ about the use of mucinous carcinoma neoadjuvant chemotherapy to overcome mucinous carcinoma resistance to chemotherapy and anti-HER2 therapy, the clinical response, reflected by changes in tumor size, was not ideal because the large volume of mucin may lead to the overestimation of tumor size, which refers to the true cancerous component. However, the amount of mucin should be of no apparent prognostic significance, and the nonresponse of tumor size does not mean that no survival benefit has occurred. The GeparTrio study^[26]^ indicates that although the clinical benefit is not obvious for luminal subtype cancers, patient's disease-free survival is prolonged.

In the present case, we treated the patient with chemotherapy and anti-HER2 therapy. She received four 21-day cycles of intravenous docetaxel (75 mg/m^2^), plus intravenous cyclophosphamide (600 mg/m^2^) and intravenous trastuzumab 8 mg/kg (loading dose) on day 1 followed by 6 mg/kg every 3 weeks to complete a full year. For endocrine therapy, our results were similar in terms of generally better efficacy when using an aromatase inhibitor compared with tamoxifen in low-risk postmenopausal women from the BIG 1–98 trial.^[27]^ Therefore, for lymph node-negative mucinous carcinoma, we can choose either tamoxifen or letrozole. However, HER2-overexpression is associated with resistance to tamoxifen,^[28]^ and PR loss is linked to poor prognosis that benefits less from tamoxifen in both adjuvant and metastatic settings.^[29]^ As a result, we selected letrozole as the treatment.

## 4 Conclusions

It is important to correctly diagnose the ER(+), PR(−), HER2(+) subtype of mucinous carcinoma. Since the ER(+), PR(−) subtype has poor prognosis,^[30]^ and HER2 positivity is associated with poor prognosis and tamoxifen resistance, this type should be treated with chemotherapy, anti-HER2 therapy, and endocrine therapy via an aromatase inhibitor.

## Acknowledgments

The authors thank LetPub (www.letpub.com) for its linguistic assistance during the preparation of this manuscript.

## Author contributions

XXX.
